# The value of lung ultrasound in the differential diagnosis of common lung diseases in newborns

**DOI:** 10.1097/MD.0000000000040459

**Published:** 2024-11-08

**Authors:** Jiabo Wu, Chang Su, Yueyan Mao

**Affiliations:** a Department of Pediatrics, Linping Branch of the Second Affiliated Hospital of Zhejiang University, Hangzhou, China.

**Keywords:** lung diseases, lung ultrasound, neonate

## Abstract

To investigate the value of lung ultrasound in the differential diagnosis of common neonatal lung diseases. A total of 160 newborns with suspected lung diseases admitted to the Department of Neonatology of Linping Branch of the Second Affiliated Hospital of Zhejiang University from January 2020 to June 2023, were selected for examination. Perform lung ultrasound within 24 hours of admission for above newborns, using the final clinical diagnosis as standard. Calculate the accuracy, sensitivity, and specificity of lung ultrasound technology in the diagnosing neonatal lung diseases, and assess its value in the differential diagnosis of common neonatal lung diseases. A total of 160 newborns suspected of having lung disease were finally diagnosed with lung disease in 142 cases. The accuracy of lung ultrasound in differentiating neonatal pneumonia, respiratory distress syndrome of the newborn, meconium aspiration syndrome, transient tachypnea of the newborn, pneumothorax, atelectasis, and pulmonary hemorrhage was 96.8%, 98.1%, 98.8%, 100%, 100%, 100%, and 100%, respectively. The detection rate of lung ultrasound examination for lung disease in newborns was 85.00%, with a sensitivity of 95.77%, specificity of 77.77%, positive predictive value of 97.14% and negative predictive value of 70.0%. The consistency test kappa value between lung ultrasound findings and the final clinical diagnosis of neonatal lung diseases is 0.846. Lung ultrasound holds significant value in the differential diagnosis of common lung diseases in newborns.

## 1. Introduction

Neonatal pneumonia, respiratory distress syndrome (RDS), neonatal meconium aspiration syndrome (MAS), neonatal transient tachypnea (TTN), pneumothorax, atelectasis, pulmonary hemorrhage, etc are the most common lung diseases in early infancy.^[1]^ Neonatal lung diseases progress rapidly and their clinical manifestations lack specificity, which may lead to underdiagnosis, misdiagnosis, and treatment delays.^[2]^ Therefore, rapid diagnosis is of paramount importance in supporting treatment and improving the prognosis of newborns. For a long time, the diagnosis of neonatal lung diseases was primarily relied on X-ray examination of the lungs. However, the examination often requires moving the infant, which poses certain limitations in clinical applications.^[3]^ With the advancements in ultrasound technology, lung ultrasound has become the preferred choice for diagnosing various lung diseases due to its noninvasive, convenience, rapid, and the ability can be performed at the bedside.^[4]^ In this context, this study aimed to observe the lung ultrasound characteristics of common lung diseases in newborns, collecting information to support the application of lung ultrasound technology in the diagnosis of neonatal lung diseases.

## 2. Methods

### 2.1. Study population

Newborns suspected of having lung diseases admitted to the Department of Neonatology of Linping Branch of the Second Affiliated Hospital of Zhejiang University from January 2020 to June 2023 were selected for the study. Inclusion criteria were as follows: (1) gestational age of 28 to 40 weeks, and birth weight of 1000 to 4000 g; (2) diagnosis of lung diseases based on clinical symptoms, physical signs, laboratory examinations and X-ray examinations; (3) infants with respiratory distress. Exclusion criteria were: (1) symptoms relieved within 24 hours; (2) patients with congenital heart disease; (3) transferred or those who opted discontinue treatment; (4) suspected patients with genetic metabolic diseases; (5) presence of contraindications for ultrasound examination. This study included a total of 160 newborns suspected of having lung diseases, comprising 78 males and 82 females; the gestational age was 34.26 ± 3.89 weeks, with a birth weight of (2870 ± 280.52) g; among them, there were 52 cases of cesarean section, 108 cases of vaginal delivery, 72 preterm births, and 88 full-term births.

### 2.2. Methods of examination

All neonates underwent lung ultrasound examination within 24 hours of birth and conducted by the same experienced sonographer who had received rigorous in lung ultrasound qualification. The sonographer examined the neonates based solely on clinical symptoms, physical examination and perinatal history, without knowledge of the results of other examinations. Then, the GE Vivi IQ diagnostic ultrasound machine with a high-frequency line array probe at a frequency of 7.5 to 10 MHz was used. The newborns were positioned in a quiet supine, lateral and prone position. Each side of the lung was divided into 3 regions: the anterior region between the sternum and anterior axillary line, the lateral region between the anterior and posterior axillary line, and the posterior region between the posterior axillary line and the spine. Firstly, the transducer was placed vertically on the ribs and slid laterally from the midline along the wide axis for a vertical scan. Then, the transducer moved from top to bottom until all lung fields were examined. Subsequently, the transducer was rotated by 90°, maintaining parallel alignment with the ribs, and slid along the narrow axis to realize a parallel scan. Move the transducer from top to bottom to scan the remaining areas until all lung fields are examined. Finally, transdiaphragmatic scanning places the transducer below the xiphoid and tilts the transducer from side to side to scan the diaphragm and lower part of the lung via the liver as an acoustic window.^[5,6]^

### 2.3. Clinical diagnostic standard (excluding lung ultrasound diagnostic criteria)

The final clinical diagnosis of neonatal pneumonia, RDS, MAS, TTN, pneumothorax, atelectasis, pulmonary hemorrhage, and other diseases was based on relevant criteria outlined in “Neonatology.” This diagnosis encompassed symptoms, physical signs and other examinations. The details are as follows:

1. Pneumonia: Lower respiratory tract infections can cause severe respiratory distress in the newborn. A comprehensive medical history, series of blood cell counts, measurement of C-reactive protein, and blood cultures contribute to the diagnosis and treatment. While, Chest X-rays can show bilateral patchy opacities with or without pleural effusion. Subsequent studies such as blood and sputum cultures can identify the causative organisms.^[7,8]^
2. RDS is caused by a deficiency of pulmonary surfactant and immature lung structure. Clinical manifestations of RDS include early respiratory distress, presenting with cyanosis, grunting, retractions, tachypnea, and blood gas measurements indicating hypoxemia and acidosis. Chest X-rays display the characteristic ground glass appearance and air bronchograms.^[7,9]^
3. TTN: Clinical presentation includes rapid shallow breathing, occasional grunting, and rarely respiratory failure. Arterial blood gases shows varying degrees of hypoxemia with normocarbia or hypercarbia. Chest X-rays reveal perihilar streaking, patchy infiltrates, increased interstitial markings, and fluid within the interlobar fissures. However, the definitive diagnosis of TTN is usually made retrospectively after resolve within 1 to 5 days of supportive treatment.^[7,8]^
4. MAS is a common cause of respiratory distress in full-term newborns shortly after birth. MAS presents with marked tachypnea, grunting, retractions, and cyanosis at birth. Examination may reveal a barrel-shaped chest, and auscultation may detect rales and rhonchi. The typical chest X-ray initially shows diffuse streaky infiltrates. Over time, the lungs become hyperinflated with patchy areas of atelectasis and infiltrates amid alveolar distension.^[10]^
5. Pneumothorax: Pneumothorax usually occurs as a secondary event to an underlying disease process but may also spontaneously occur in 1% of newborns. Clinical manifestations range from mild to severe respiratory distress,with respiratory function gradually worsening. Diagnosis usually requires chest X-rays to confirm and identify underlying causes.^[7,8]^
6. Atelectasis: Atelectasis is not only an independent disease, but also a common complication of various diseases. Typically, on chest X-rays, lung collapse is characterized by increased density and decreased intercostal space, distortion of mediastinal structures such as the trachea and heart, elevated diaphragm on the affected side, and hyperinflation of the contralateral lung.^[11]^
7. Pulmonary hemorrhage is not an independent disease, but is often a late complication of other diseases. Besides respiratory tract injuries and gastrointestinal bleeding, clinical symptoms worsen significantly, and the presence of fresh blood leaking into the oral or nasal cavity or endotracheal tube serves as diagnostic criteria. Chest X-ray may reveal mottled or hazy opacities in the peripheral lung tissue, and in severe cases, pulmonary whiteness may be observed.^[12]^

Ultrasound diagnostic criteria for different lung diseases are shown in references.^[5,6,13–20]^

### 2.4. Data analyses and statistics

Statistical analyses were performed using SPSS for Windows Version 26 (IBM Corp., Armonk, NY). Statistical data are expressed as n (%), and the *χ*2 test or Fishers exact test was used for comparison between groups. Normally distributed measures are expressed as mean ± standard deviation, and the *t* test for 2 independent samples was used for comparison between groups, differences were considered statistically significant at *P* < .05.

Sensitivity = number of true positive cases/(number of true positive cases + false negative cases), specificity = number of true negative cases/(false positive cases + true negative cases), positive predicted value = positive cases/(true positive cases + false positive cases), negative predicted value = true negative cases/(false negative number + false negative cases), accuracy = (the number of true positive cases + true negative cases)/(true positive number of false positive cases + false negative cases + true negative cases).

## 3. Results

1. Final clinical diagnoses of 160 neonates with suspected lung diseases: Among them, there were 142 cases of pulmonary diseases, including 70 cases of pneumonia, 37 cases of RDS, 10 cases of MAS, 10 cases of TTN, 4 cases of pneumothorax, 7 cases of atelectasis, and 4 cases of pulmonary hemorrhage.
2. After lung ultrasound examinations for 160 newborns with suspected pulmonary diseases, 136 neonates were diagnosed as positive. It yielded a positive detection rate of 85.0% (136/160) for neonatal lung diseases, with a sensitivity of 95.77% (136/142), specificity of 77.77% (14/18), positive predictive value of 97.14% (136/140), and negative predictive value of 70.0% (14/20). The Kappa value for consistency testing was 0.846, indicating a high level of agreement between the 2, as shown in Table 1.
3. The ultrasound diagnostics and radiological characteristics of 160 newborns with suspected lung disease. The lung ultrasound diagnosed pneumonia in 66 cases with an accuracy rate of 96.8%. All patients showed pulmonary consolidation with the disappearance of the A-lines and the appearance of B-lines. 36 cases diagnosed with RDS through lung ultrasound, with an accuracy rate of 98.1%. All cases showed pulmonary consolidation with the disappearance of the A-lines, pleural line abnormalities, and changes consistent with alveolar interstitial syndrome. Nine cases were diagnosed with MAS through lung ultrasound, with an accuracy rate of 98.8%. The affected areas in all cases showed the disappearance of A-lines, focal subpleural consolidations, changes consistent with alveolar interstitial syndrome, and abnormal pleural line alterations. However, these changes were deemed nonspecific. Then, lung ultrasound diagnosed TTN in 10 cases, with a 100% accuracy rate. All cases exhibited alveolar interstitial syndrome, abnormal pleural lines, and the disappearance of A-lines, with no pulmonary consolidation. The ultrasound diagnosis of pneumothorax was made in 4 cases, with a 100% accuracy rate. All cases showed the disappearance of lung sliding absence of B-lines, and M-mode imaging showed “barcode or stratospheric sign” and “lung dot” were found. Lung ultrasound diagnosed pulmonary atelectasis in 7 cases with a 100% accuracy rate. All cases showed a large consolidated area in the lungs accompanied by bronchial dilation, clear boundaries, and disappearance of A-lines and abnormal pleural lines. Lung ultrasound diagnosed pulmonary hemorrhage in 4 cases with a 100% accuracy rate. All cases showed signs of lung consolidation, bronchial inflation, abnormal pleural lines, disappearance of A-lines and alveolar interstitial syndrome. Therefore LUS demonstrated a high diagnostic accuracy for different respiratory disorders among the studied neonates, as presented in Table 2.

**Table 1:** Comparison of the results of lung ultrasound diagnosis and final clinical diagnosis in 160 newborns with susception.

Lung ultrasound	Clinical diagnostic results		Total	Kappa
	Negative	Positive		
Positive	4 (2.5%) 14 (8.8%) 18 (11.3%)	136 (85%) 6 (3.7%) 142 (88.7%)	140 (87.5%)	
Negative			20 (12.5%)	0.846
Total			160 (100%)	

**Table 2:** Sensitivity, specificity, and accuracy regarding LUS in studied respiratory diseases.

Diagnosis (N)	TP	TN	FP	FN	Sensitivity	Specificity	Accuracy
RDS (38)	36	121	1	2	94.7%	99.2%	0.981
Pneumonia (69)	66	89	2	3	95.6%	97.8%	0.968
TTN (10)	10	150	0	0	100%	100.0%	1.000
MAS (10)	9	149	1	1	90%	99.3%	0.988
PNX (4)	4	156	0	0	100%	100%	1.000
Atelectasis (7)	7	153	0	0	100%	100%	1.000
Pulmonary hemorrhage (4)	4	156	0	0	100%	100%	1.000

FN = false negative, FP = false positive, MAS = meconium aspiration syndrome, N = number of patients, PNX = pneumothorax, RDS = respiratory distress syndrome, TN = true negative, TP = true positive, TTN = transient tachypnea of newborn.

## 4. Discussion

Various pulmonary diseases with different etiologies are common in newborns, constituting a major cause of hospitalization and death.^[21]^ Timely and accurate diagnosis is crucial for proper treatment and improving prognosis. In the past, X-rays and CT scans were utilized; however, chest X-rays and CT scans have notable limitations, especially considering the increased risk and potential DNA damage associated with CT scans in newborns.^[22]^ Our research reveals that lung ultrasound examination in newborns achieved a positive detection rate of 85.00%, with a sensitivity of 95.77%, specificity of 77.77%, positive predictive value of 97.14%, and the negative predictive value of 70.0%. The consistency test (kappa value) between the lung ultrasound findings the final clinical diagnosis of newborn pulmonary diseases was 0.846. The accuracy of lung ultrasound in distinguishing and diagnosing neonatal pneumonia, RDS, MAS, TTN, pneumothorax, atelectasis, and pulmonary hemorrhage was 96.8%, 98.1%, 98.8%, 100%, 100%, 100%, and 100%, respectively. This suggests that lung ultrasound holds high value in the differential diagnosis of pulmonary diseases in newborns.^[23]^

The sensitivity of LUS in diagnosing RDS was 94.7%, with a specificity of 99.2% and an overall diagnostic accuracy of 98.1%. These results were similar to the findings of Corsini et al, who reported a sensitivity of 94% and specificity of 100%, respectively.^[24]^ Our study identified pulmonary consolidation, disappearance of A-lines, abnormal pleural lines, and alveolar interstitial syndrome as the main diagnostic features of RDS using LUS. This is consistent with the conclusions drawn by Chen SW et al.^[25]^

For pneumonia diagnosis, LUS demonstrated a sensitivity of 95.6% and specificity of 97.8%, with an overall diagnostic accuracy of 96.8%. These results may be similar to those reported by Ismail R et al, with sensitivity of 97.5% and specificity of 95%.^[26]^ Nevertheless, Corsini et al confirmed 100% sensitivity and specificity.^[24]^ The LUS features for pediatric and neonatal pneumonia are similar, primarily characterized by signs of bronchial inflation, shredding sign (100% of cases), pulmonary consolidation (100% of cases), and abnormal pleural lines, with or without pleural effusion.

In the diagnosis of TTN, LUS demonstrated a sensitivity and specificity of 100%, with an overall diagnostic accuracy of100%.This aligns with the results reported by Corsini et al, who confirmed a sensitivity and specificity of 100% and 98%, respectively.^[24]^ Our study found that the disappearance of the A-lines and the absence of pulmonary solid lesions, double lung point, were LUS’s main TTN diagnostic features. In our research, 100% of TTN cases presented with double lung point, which is characterized by a rapid increase in echo intensity in the lower lung fields of neonates with TTN and is considered a diagnostic feature.^[27,28]^ Moreover, we found that the primary ultrasonic feature of RDS cases was pulmonary consolidation with air bronchogram signs but without double lung point. In contrast, L Jing et al reported the same results, indicating that LUS can easily distinguish between TTN and RDS.^[28]^

In the diagnosis of MAS combined with LUS, this study demonstrated a sensitivity and specificity of 90% and 99.3%, respectively, with an overall diagnostic accuracy of 98.8%. This is consistent with the findings of Liu et al, confirming LUS as a reliable method for diagnosing MAS.^[28]^ We identified the main diagnostic features of MAS using LUS as the disappearance of A-lines, focal subpleural solid lesions, changes consistent with interstitial-alveolar syndrome, and abnormal pleural line alterations. However, all these changes were nonspecific, resembling LUS findings in pneumonia. Similar results were reported by Piastra et al.^[20]^

In the diagnosis of pneumothorax using LUS, this study demonstrated a sensitivity and specificity of 100% and 100%, respectively, with an overall diagnostic accuracy of 100%. This is likely similar to the results reported by Corsini et al who reported a sensitivity of sensitivity of 80% and a specificity of 100%.^[24]^ In contrast, Lichtenstein et al, reported a sensitivity of 79% and a specificity of 100%.^[29]^ In all cases, lung sliding was absent, B-lines were not observed, and M-mode imaging showed a “barcode” or “stratosphere sign.”

The current results indicate a sensitivity and specificity of 100% each for the diagnosis of pulmonary atelectasis using LUS. With an overall diagnostic accuracy of 100%. These sensitivity results align with those reported by Liu et al, which also demonstrate regarding 100% sensitivity; however, their specificity was 75%.^[30]^ Furthermore, Acosta CM et al reported a 100% sensitivity for LUS in diagnosing pulmonary atelectasis in children.^[31]^ In all cases, there was extensive pulmonary consolidation with air bronchograms, disappearance of A-lines, and abnormal pleural lines.

In addition, the current results indicate a sensitivity and specificity of 100% each for the diagnosis of pulmonary hemorrhage in newborns using LUS showed with an overall diagnostic accuracy of 100%. All cases showed sign of shredding, bronchial inflation, abnormal pleural lines, and the disappearance of A-lines, indicative of changes consistent with alveolar interstitial syndrome. However, pulmonary hemorrhage is not an independent disease; its diagnosis requires comprehensive clinical examinations.^[15]^

The limitation of this study include the fact that the conclusions are based on a small number of cases, particularly in the instances of pulmonary atelectasis pulmonary hemorrhage and pneumothorax. The case numbers are limited, lacking statistical power. Additionally, this study was conducted at a single center, utilizing a specific ultrasound machine, and the ultrasound results have not been validated by other experts. Therefore, methodological rigor and multicenter studies are needed to confirm these findings and further assess the performance of lung ultrasound in diagnosing common lung diseases in newborns.

## 5. Conclusions

In conclusion, application of ultrasound has significant value in the clinical application of common lung diseases in newborns. It demonstrates high sensitivity, specificity and accuracy, is noninvasive, bedside-accessible, available for real-time monitoring and allows for dynamic observation. It possesses advantages such as accuracy, reliability, avoidance of radiation damage, and low-cost, making it particularly important. Therefore, its promotion is warranted. However, lung ultrasound has not yet replaced X-ray examinations. Given the complexity of clinical conditions, a comprehensive examination, including medical history, is still necessary.

## Acknowledgments

We are very grateful for the support and help of ultrasound doctors and colleagues.

## Author contributions

**Data curation:** Chang Su.

**Formal analysis:** Chang Su.

**Methodology:** Jiabo Wu, Yueyan Mao.

**Project administration:** Jiabo Wu, Yueyan Mao.

**Resources:** Jiabo Wu, Yueyan Mao.

**Software:** Yueyan Mao.

**Writing – original draft:** Jiabo Wu.

**Writing – review & editing:** Jiabo Wu, Chang Su.
